# Preventive Legislation in Forensic Medicine*Read at 36th annual meeting State Medical Association of Texas, Austin, Texas, April 28, 1904.Contributed to the Society on the Invitation of its Committee.

**Published:** 1904-09

**Authors:** Clark Bell

**Affiliations:** The New York Bar


### TEXAS MEDICAL JOURNAL.

ESTABLISHED JULY, 1885.

PUBLISHED MONTHLY.—SUBSCRIPTION $1.00 A YEAR.

VOL. XX. AUSTIN, SEPTEMBER, 1904. No. 3.

### Original Contributions.

For Texas Medical Journal.

### Preventive Legislation in Forensic Medicine.*

BY CLARK BELL, ESQ., LL. D., OF THE NEW YORK BAR.

There can be no question of the right of the government of a State acting through its constitutional powers by legislative enactment, to adopt and pass such laws, rules and regulations as will inure to the welfare of its citizens in averting the spread of an infectious disease.

In every American State this power of the State by suitable legislation to protect its inhabitants from an invasion of an epidemic which threatens the health or the lives of its people, rests on as solid a foundation as would the power of the State to resist invasion by a public enemy threatening with an army of soldiers the lives and the property of the people of a State.

The theory of government under which our American Constitutions were formed was that governments were founded in a paternal sense to protect the citizen in his rights, and to defend him when the danger became a public menace to life, limb, or health.

This principle has been recognized in our American States and by our general government to the fullest extent by the adoption and enforcement of such sanitary legislation as the public author-

*Read at 36th annual meeting State Medical Association of Texas, Austin, Texas, April 28, 1904.

Contributed to the Society on the Invitation of its Committee.

ities under forms of law and by legislative authority deemed necessary for the public health and welfare. The whole system of quarantine regulations adopted by our general government and by our several States that border on our enormous sea coast, is based upon this principle,—that the right of the individual citizen must yield when the peril involves the many.

Cholera, yellow fever, and smallpox illustrate, in their historical relation to our people in the past, how far the whole power of the government has been called forth and exercised in the enforcement of measures, restrictions and regulations deemed essential for the welfare of communities, and in disregard of the private rights of individuals.

To justify, however, the exercise of such enormous power by legislatures as to make preventive legislation effectual and a public safeguard, especially such as contained provisions so drastic and stringent as has been found oftentimes necessary in the strict enforcement of quarantine regulations, sometimes apparently oppressive, especially those which ignored the private rights of the citizen not only, but actually, superseded and trampled upon those rights guaranteed by the organic laws; certain prerequisites are necessary and fundamental.

Is the disease either contagious, infectious or communicable, and is this such a condition as places the general safety of the public in peril unless its spread is averted?

If a man or his wife develop smallpox, beyond all question, cavil or doubt, it is the right of the State to enter the home, and, ignoring the personal rights, take the father from the bosom of his family, or the wife from the arms of her husband, and confine and segregate them. It rests on the principle that the public welfare is higher and more sacred than the rights of the individual citizen when the former is in peril.

The courts of our States sustain this extraordinary subversion and overthrow of the constitutional rights of the citizen where the presence of the disease and danger to the public is so imminent that it has become a menace to the health of the whole people. We are prone sometimes to condemn what is oftentimes an apparent private calamity to an individual, or to isolated groups of individuals when the law compels us to close our eyes to the sufferings of a few made indispensably necessary for the protection of the many, by means of measures and legislation which we have styled "preventive," both as to legislation and to medical treatment.

If, by prompt, instantaneous action we fence out the cholera from a great city like New York, or the yellow fever or smallpox from Galveston or New Orleans, we avert untold misery to thousands who thus escape contagion and avoid the appalling consequences which result inevitably if the precautionary measures and steps had not been taken and strictly, and even harshly, enforced.

The human race at this moment has probably no more terrible an adversary to contend with in the whole catalogue of diseases to which mankind is liable than this dread, this terrible disease. It is a scourge. Its ravages are indescribable, incalculable, unmeasurable. Its victims are of our most beautiful women, our most gifted men.

Until quite recent years it has been pronounced by the whole medical faculty an incurable disease.

The medical man, ten years ago, who would have dared to announce that he could cure it would have been denounced as a quack, and kicked out of the profession by general consent. Mankind withstood its ravages in stolid despair. Its fatalities fell like the rain on the whole race, and whoever felt it on his face prepared for death. It was pitiless because remediless, and its victims are the countless millons, yes, myriads of myriads, of the unmeasurable past.

In the problem now confronting civilization, the crucial question on which the whole fabric of preventive legislation must be built, if it is to be erected at all, is this: Is consumption a communicable disease from one human being to another? Can any plan or means be devised by which its ravages can be averted, or even arrested? How far is it within the control of legislative action by means of preventive legislation?

The first question which, on its face, might be supposed to be a medical question, on a more careful examination can not be so regarded. It is not a purely medical question. Medical men as such would not be united on an answer to it, but they are now more nearly unanimous than ever before.

It requires in its solution a higher scientific knowledge of the pathology and bacteriology of the disease than that possessed by the average medical men of our day.

But it steadily has sufficiently advanced since the researches and discoveries of Koch, and the discussions regarding the same, that there is now a belief in the mind of a large preponderance of medical men, based on the result of the researches of the ablest scientific students and observers of the subject, considered in its pathological and bacteriological aspect, that it is communicable disease.

Dr. Henry B. Baker, so long Secretary and General Executive Manager of the State Board of Health of Michigan, prior to the first session of the American Congress on Tuberculosis, in reply to my question, "What has been, in your opinion, the most notable advance in that branch of forensic medicine with which you are familiar during the 19th century?" replied as follows: "The most notable advance in forensic medicine in the 19th century is the aid which the law gives to the restriction of that disease that causes most deaths." One reason why this is true is that because that disease which causes most deaths is now known to be a preventable disease.

In 1893 the Michigan State Board of Health passed a resolution, "That hereafter consumption (and other diseases due to the bacillus tuberculosis) shall be included in the official list of 'diseases dangerous to public health,' referred to in the law requiring notice by householders and physicians to the local health officer as soon as such disease is recognized."

Judge Abram H. Dailey, ex-President of the Medico-Legal Society, and a conscientious and diligent student of medical jurisprudence, replied to the same question as follows: "In my opinion that which has prevented the inception and spread of disease through sanitary laws based upon the causes of disease and removal of the same is the most notable."

The more advanced thinkers and observers among medical men are coming to regard consumption as a curable disease if taken early in hand, despite the almost universal recent belief of the profession to the contrary.

A still larger class of medical men of higher attainments class it as a disease where preventive means are justifiable.

When the American Congress on Tuberculosis was organized in 1900 it was only the more advanced observers and thinkers who would have asserted that the disease was infectious and communicable, not the majority of the profession. While the bacillus tuberculosis was definitely determined, recognized and acknowledged by the advanced students, we could not then have said that this was the unanimous belief of medical men everywhere.

Again, it would not quite answer to accept the beliefs and opinions of medical men as such as final or even conclusive. Suppose they had, as the result of their reading and consultation among themselves, stated that they accepted it. That would establish nothing. It was not based upon the original research of any of them.

Few medical men as such have investigated the subject and studied the tubercle bacillus with the microscope and the aids that science now lends to the study and worked the problem out for themselves. Courts would not hold them to be competent witnesses as to the facts which the skilled observers had reached and recorded as scientific facts by competent observers, by original research.

That the whole subject was a proper field of scientific and legislative inquiry as to how far preventive measures could be utilized to arrest the spread of the disease and avert its awful ravages, would now be conceded by a majority of that same profession in every State in the American Union, who ten years ago, or fifteen, believed it to be incurable, and who had not made up their minds to accept the discovery which was first announced by Koch in 1882. Those who had accepted Koch's theories in the beginning, or many of them, had been all at sea by the announcements he made at the London Congress which was completely unprepared for his paper or to meet it in any satisfactory way, at that time, and I am not prepared to say that the doubts he raised have been dissipated.

The question of the hour, the problem which confronts us at the American International Congress on Tuberculosis at the Universal Exposition at St. Louis next fall is: How far can preventive legislation be of service in securing beneficial results for mankind in diminishing the volume of the disease, in either averting or arresting its terrible advances?

The problem is one of profound and intense human interest. It is a question lying wholly within the domain of medical jurisprudence.

The government of the United States in its splendid sympathetic and paternal action, sent to the officers of the management of the St. Louis Congress of 1904, in the language it employed in its instructions to the American Diplomatic Corps sent to foreign governments respecting the aims and purposes of this Congress, was as follows:

"The humanitarian object which this Congress has in view to reach, by the discussion of scientific men, some result in arresting the spread and averting, so far as it may be found possible, the ravages of this dreadful disease, which now falls with such terrible force and fatality upon the people of the Western Hemisphere, can not but enlist the sympathy and approval of the government to which you are accredited."

Texas will hold a high position in the labors and councils of the St. Louis Congress. It will be represented by the ablest names of the profession in the Lone Star State.

Texas already holds four places on the Committee of Organization named by the World's Fair Exposition, and Texas will do her part well at St. Louis.

It is a question higher than that of medical schools or medical politics or difference of opinion as to theories or schools. It needs in its solution the ablest minds of your State, not only in law and in medicine, but in all the professions, for such a solution as shall be worthy of the aims so admirably presented by the State Department of the American Government which I have quoted.

I have yielded to the request of your committee to say a few words to you, which I submit in the hope that they may encourage medical men of Texas to aid us in our humane endeavor for a common good to the race in the struggle now going forward with this dread disease by the scientific world and in which the medical men of your State feel so deep and profound an interest.

